# Comprehensive bibliometric and visualized analysis of research on fecal microbial transplantation published from 2000 to 2021

**DOI:** 10.1186/s12938-022-01046-y

**Published:** 2022-10-29

**Authors:** Jing Ma, Ting Chen, Xiangxue Ma, Beihua Zhang, Jiaqi Zhang, Lin Xu, Yifan Wang, Jinke Huang, Zhihong Liu, Fengyun Wang, Xudong Tang

**Affiliations:** 1 Institute of Digestive Diseases, Xiyuan Hospital of China Academy of Chinese Medical Sciences, Beijing, 100091 China; 2 Department of Gastroenterology, Shenzhen Hospital of Traditional Chinese Medicine, Shenzhen, China; 3 Department of Gastroenterology, Peking University Traditional Chinese Medicine Clinical Medical School (Xiyuan), Beijing, China

**Keywords:** Fecal microbial transplantation, Bibliometric, Visualized analysis, Biblioshiny, CiteSpace

## Abstract

**Background:**

Fecal microbial transplantation has emerged in recent years as a method of treating disease by rebuilding the intestinal flora. However, few bibliometric analyses have systematically studied this area of research. We aimed to use bibliometric analysis to visualize trends and topical research in fecal microbial transplantation to help provide insight into future trends in clinical and basic research.

**Materials and methods:**

Articles and reviews related to fecal microbial transplantation were collected from the Web of Science Core Collection. Significant information associated with this field was visually analyzed by using Biblioshiny and CtieSpace software.

**Results:**

A total of 3144 articles and overviews were included. The number of publications related to fecal microbial transplantation significantly increased yearly. These publications mainly came from 100 countries, led by the US and China, and 521 institutions. The most prolific and influential author is KHORUTS A. The main disciplines and application fields of fecal microbial transplantation included molecular /biology/immunology and medicine/clinical medicine, and the research foundation of fecal microbial transplantation was molecular /biology/genetics and health/nursing/medicine. An alluvial flow visualization showed several landmark articles. New developments were identified in terms of reference and keyword citation bursts. Data analysis showed that different FMT preparation and delivery methods gradually appeared as research hotspots. The main research keywords in the last 3 years were chain fatty acids, *Akkermansia muciniphila*, and insulin sensitivity, other keywords were current and developing research fields.

**Conclusion:**

Research on fecal microbial transplantation is flourishing and many new applications of fecal microbial transplantation are emerging. Microbial metabolites such as short-chain fatty acids and the microbiota–gut–brain axis have become the focus of current research and are future research trends.

## Introduction

The gastrointestinal tract of mammalian is an ideal habitat for various microorganisms, such as bacteria, fungi, viruses and archaea [1, 2]. The gut contains a complex ecosystem of trillions of commensal microbes [3], the intestinal mucosa, where the microbiota and immune system engage in extensive bidirectional communication, is the best-studied interface for host–microbiota interactions [4]. Growing evidence demonstrates that the microbiota and derived microbial compounds play a pivotal role in the development and maintenance of normal intestinal development and physiology, including host immunity, food digestion, tissue development and the regulation of gut endocrine function and neurological signaling [5]. Most of the microorganisms that inhabit the gut are affected by the method of birth, breastfeeding, daily lifestyle, drugs and genes of the host [6]. With exposure to environmental factors, the gut microbiota matures gradually during childhood and remains relatively stable during late childhood, puberty and adulthood [7, 8]. Maturing gut microbe core functions include genes encoding glycosaminoglycan degradation; short-chain fatty acids (SCFAs) production through complex polysaccharide fermentation and, specifically lipopolysaccharide (LPS) synthesis; and the biosynthesis of some critical amino acids and vitamins [9–12]; whereas, reciprocal interactions between the gut microbiota and host immunity are complicated, dynamic and environment dependent [4]. The use of antibiotics, changes in diet and geographic location can disrupt the host–microbiome interface, and damage to the immune system can lead to systemic dissemination of commensal microorganisms, susceptibility to pathogen intrusion, and abnormal immune reactions.

Fecal microbial transplantation (FMT) is the process of transferring minimally handled prescreened donor feces into a patient's gastrointestinal tract with the aim of correcting dysbiosis by increasing overall diversification and restoring microbiota functions [13]. In 2013 [14], a randomized controlled clinical trial (RCT) showed that FMT was generally safe and highly effective in the treatment of recurrent *Clostridioides difficile* infection (RCDI), led FMT to become an intriguing but poorly understood intervention in a prevailing condition of global concern. Currently, the only established indication for FMT is CDI [15]. Inflammatory bowel disease (IBD) may be an emerging indication for FMT. To date, four RCTs have been published investigating the use of FMT for the treatment of ulcerative colitis (UC) [16–19]. Gut microbes not only shape the function of the gut but also influence the physiological function of other important extraintestinal organs, including the liver, lungs and brain [2], so there are many future directions and areas of uncertainty, such as hepatic encephalopathy (HE) [20, 21], primary sclerosing cholangitis (PSC) [22], metabolic syndrome and obesity [23, 24], and neurological disorders such as Parkinson's disease [25, 26] and autism spectrum disorders [27, 28].

Microorganisms that reside in the gastrointestinal tract (GIT) play a critical role in the maturation and development of the immune system, central nervous system and digestive system [29]. Many diseases occur with intestinal microorganism imbalances. FMT can restore intestinal homeostasis and structure, so it is widely used in the treatment of many diseases. However, up to now, no articles have systematically studied FMT using bibliometrics.

Bibliometric analysis is a comprehensive knowledge system focusing on quantification, and has been widely used to gain insight into scientific development trends. Through bibliometric analysis scholars cannot only quickly grasp the research hotspots and development trends of a specific research field, but also evaluate the distribution of countries, authors in the research field. Web of Science Core Collection is a common database used in bibliometric analysis. To analyze the active countries and authors on FMT, the R-bibliometrix package (version 3.0.3, http://www.bibliometrix.org) in R-Studio (version 1.2.1335) was used. Biblioshiny, a shining app provides bibliometrics was used to support the import of metadata from database and subsequent data management [30]. CiteSpace which is focused on the analysis of the potential facts contained in the scientific literature was used to analyze the subject categories, citation bursts of references and keywords, and the alluvial flow visualization of co-cited references [31].

## Results

### Trends in publications

**Fig. 1:**
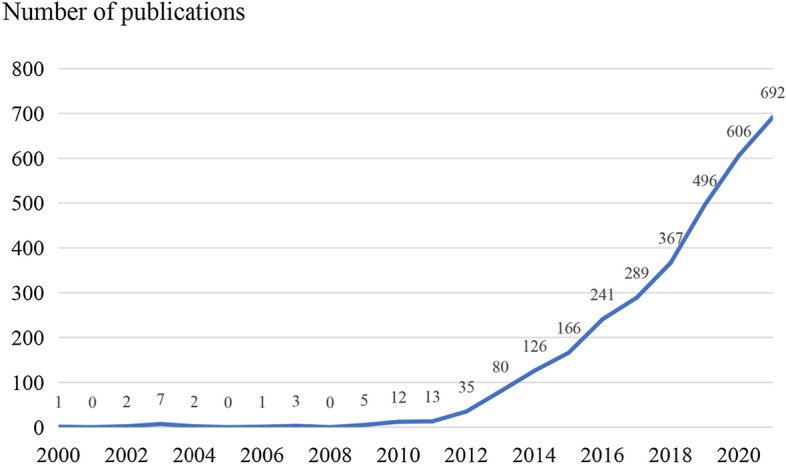
Trend of publications in fecal microbial transplantation from 2000 to 2021

Annual literature publications in a specific field can directly reflect the development of that field. Figure 1 reflects that the research on FMT before 2009 showed a fluctuating trend; since 2010, research on FMT is increasing in the literature. The period from 2000 to 2009 was an embryonic period. From 2010 to 2014, publications on FMT showed a continuous growth trend, during which the highest annual growth rate of published papers was approximately 169.2% (2011–2012). From 2015 to 2021, the annual publication growth rate was relatively stable, and the highest number of articles published reached 692 per year, indicating that FMT as an emerging therapy may become a research hotspot for basic and clinical research.

### Active countries/regions and authors

**Fig. 2:**
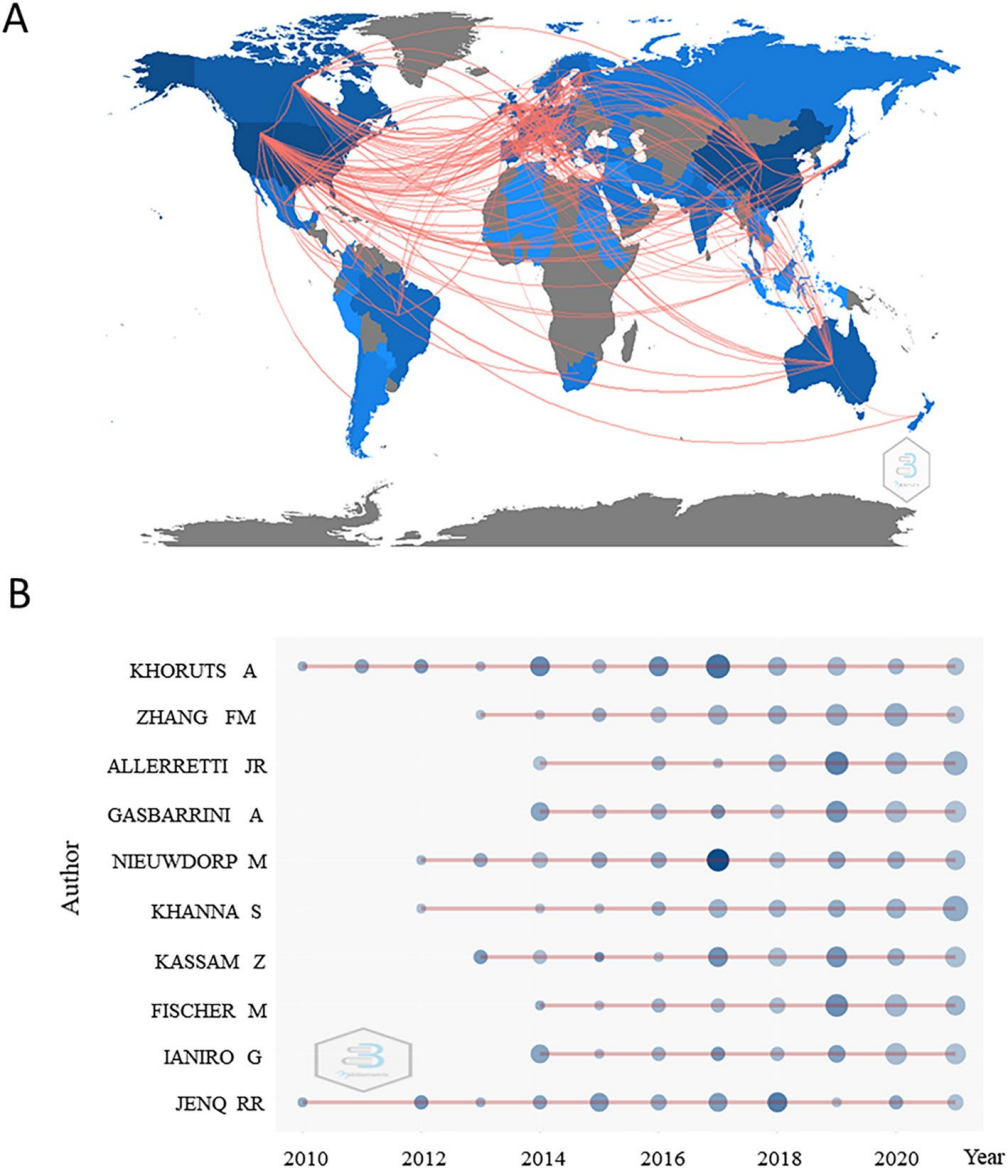
Analysis of active countries and authors. **A** Country collaboration map of studies associated with fecal microbial transplantation. **B** The top ten active researchers in the field of fecal microbial transplantation and their publications over time. Size of circle represents number of publications, and the deeper the color, the more citations

A total of 3144 articles were published by 521 institutions in 100 countries. Tables 1 and 2 summarize top 10 countries and institutions based on their total publications, year in tables represent the first posting time of relevant literature in country or institution. Centrality of a node in the network measures the importance of the position of the node in the network. Citespace uses this metric to identify and measure the importance of an article, highlights such literature (or authors, journals, institutions, etc.) with purple circles and nodes with centrality over 0.1 called critical nodes. Table 1 shows that the United States (US) was the most productive country, with the highest centrality of 0.24, followed by China and Italy. Table 2 shows that Harvard Med School was the institution with the most productive country, however its centrality was relatively low when compared to other institutions. By contrast, the University of Minnesota System, University of Michigan and University of Alberta had relatively higher centrality. Figure 2A displays the global country cooperation map produced through the application of the biblioshiny app. There were 1101 pairs of collaborating countries/regions worldwide, and most pairs came from the US, whose total of 501 pairs reflect its close cooperation with the other 14 countries. The top 10 most prolific researchers are shown in Fig. 2B, and the most productive author was Khoruts A, who is from the University of Minnesota, followed by Zhang FM, pioneer of FMT in China. Table 3 shows the information about the outstanding researchers in FMT.

**Table 1:** Top 10 countries ranked by total publications

No.	Country	Year	Centrality	TP (%)
1	USA	2007	0.24	1170 (37.21%)
2	Peoples R China	2011	0.00	789 (25.10%)
3	Italy	2013	0.03	185 (5.89%)
4	Germany	2009	0.06	179 (5.69%)
5	Canada	2009	0.05	178 (5.66%)
6	France	2010	0.17	155 (4.93%)
7	England	2010	0.07	154 (4.90%)
8	Netherlands	2009	0.01	133 (4.23%)
9	Australia	2010	0.03	121 (3.85%)
10	Japan	2012	0.01	103 (3.28%)

*TP* total publications

**Table 2:** Top 10 institutions ranked by total publications

No.	Institution	Year	Centrality	TP (%)
1	Harvard Med School	2016	0.02	77 (2.45%)
2	University of Minnesota System	2010	0.14	74 (2.35%)
3	Mayo Clinic	2012	0.08	63 (2.00%)
4	Nanjing Medical University	2013	0.01	61 (1.94%)
5	Zhejiang University	2011	0.04	58 (1.84%)
6	Mem Sloan Kettering Canc Ctr	2010	0.04	55 (1.75%)
7	University of Michigan	2013	0.12	49 (1.56%)
8	University of Amsterdam	2009	0.06	46 (1.46%)
9	University of Helsinki	2013	0.05	44 (1.40%)
10	University of Alberta	2009	0.14	43 (1.37%)

*TP* total publications

**Table 3:** Top 10 active researchers ranked by their total publications

No.	Authors	TP	TC	ACPP	H-index
1	Khoruts A	48	4946	103.04	27
2	Zhang FM	40	1070	26.75	19
3	Allegretti JR	38	1105	29.08	16
4	Gasbarrini A	38	1678	44.16	17
5	Nieuwdorp M	38	3147	82.82	23
6	Khanna S	37	1049	28.35	19
7	Kassam Z	35	2539	72.54	19
8	Fischer M	33	1207	36.58	14
9	Ianiro G	31	1625	52.42	15
10	Jenq RR	31	4130	133.23	24

*TC* total citations, *ACPP* average citation per paper

### Subject categories

**Fig. 3:**
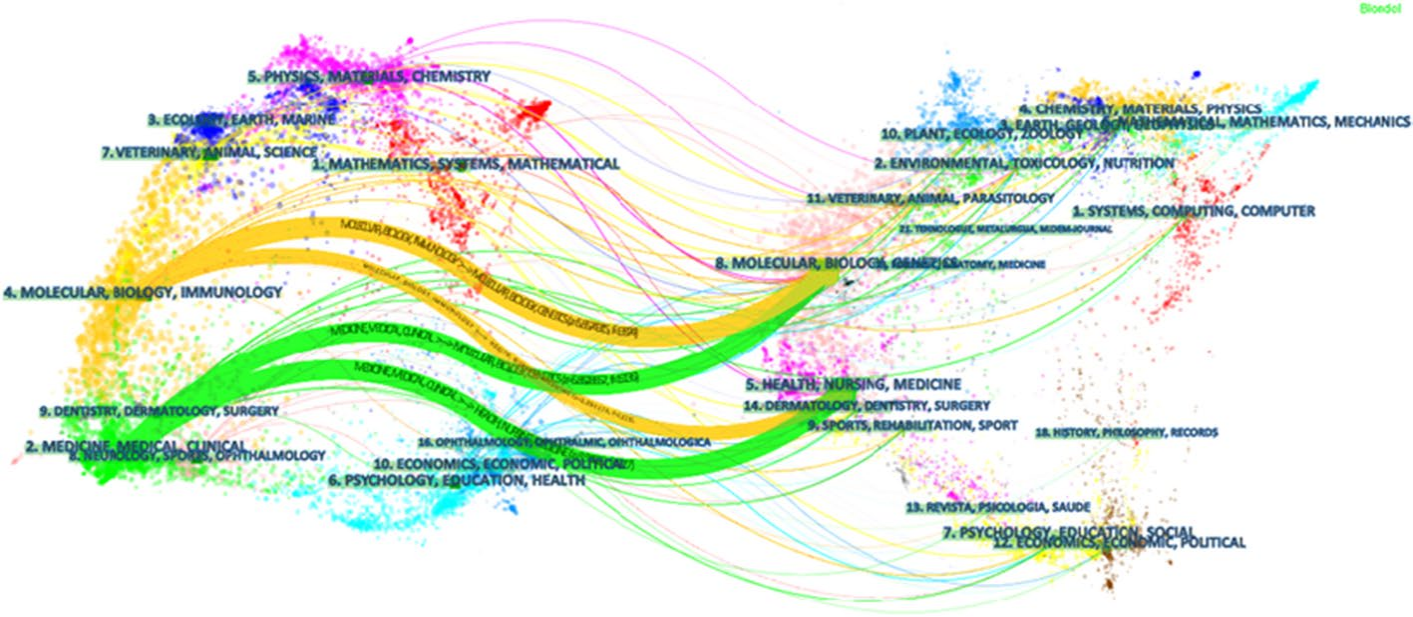
The dual-map overlay of journals on fecal microbial transplantation

A dual-map overlay for the journals can reflect the subject categories of FMT and the areas in which FMT is most applied. In Fig. 3, on the left is the distribution of the cited literature in the journals representing the main disciplines and application fields of FMT, which included molecular/biology/immunology and medicine/clinical medicine; on the right is the distribution of the corresponding cited literature in the journals representing the main disciplines citing FMT and the research foundation, which included molecular/biology/genetics and health/nursing/medicine. The colored curves between them are citing paths, which fully show the cited relationships.

### Bursts of cited references

The rapid development of a field is characterized by articles that experience a citation burst [32]. Table 4 shows the top 10 references with the strongest citation bursts during 2000–2021. The article with the strongest citation burst was published in The New England Journal of Medicine by van Nood E in 2013, and its burst continued for 5 years, with a burst strength of 101.51 [14]. The article with the second-strongest burst was by Gough E in 2011, with a burst strength of 66.95 [33]. Table 5 shows the top 10 references with the strongest citation bursts during 2019–2021. Among the articles with strong citation bursts, McDonald LC’s 2018 article [34], which was published as a clinical practice guideline for CDI in adults and children, had the strongest citation burst with a burst strength of 52.81. The second article with the most recent citation burst reported the effect of FMT on 8-week remission in patients with UC [19]. This work demonstrated that adults with mild to moderate UC were more likely to be in remission at 8 weeks if they received 1 week of anaerobically prepared donor FMT than if they received autologous FMT. The third article that has drawn much attention was a 2018 article published in Science [36] which found that dysbiosis in gut microbiome composition may result in primary resistance to immune checkpoint inhibitors and proved that the gut microbiome affects the efficacy of PD-1-based immunotherapy for epithelial tumors. The fourth article was a systematic review with a meta-analysis [37] that provided evidence that FMT is an effective treatment for RCDI. The fifth article was a randomized clinical trial about whether the clinical efficacy of FMT for RCDI differed according to the route of delivery, and the results showed that among patients with RCDI, FMT via oral capsules was comparable to colonoscopy in preventing recurrent infection over 12 weeks, suggesting that oral capsule therapy may be a convenient method of treating RCDI [38].

**Table 4:** Top 10 references with the strongest citation bursts during 2000–2021

		Citation burst			
Rank	References	Strength	Begin	End	Duration
1	van Nood E, 2013, NEW ENGL J MED, V368, P407 [14]	101.51	2014	2018	
2	Gough E, 2011, CLIN INFECT DIS, V53, P994 [33]	66.95	2012	2016	
3	McDonald LC, 2018, CLIN INFECT DIS, V66, P0[34]	52.81	2019	2021	
4	Bakken JS, 2011, CLIN GASTROENTEROL H, V9, P1044 [35]	52.36	2012	2016	
5	Moayyedi P, 2015, GASTROENTEROLOGY, V149, P102 [17]	49.51	2016	2021	
6	Cammarota G,2017, GUT, V66, P596 [36]	47.74	2018	2021	
7	Paramsothy S, 2017, LANCET, V389, P1218[18]	47.34	2018	2021	
8	Kassam Z, 2013, AM J GASTROENTEROL, V108, P500 [40]	43.72	2014	2018	
9	Costello SP,2019, JAMA-J AM MED ASSOC, V321, P156 [19]	43.46	2019	2021	
10	Surawicz CM,2013, AM J GASTROENTEROL, V108, P478 [41]	39.3	2014	2018

**Table 5:** Top 10 references with the strongest citation bursts during 2019–2021

		Citation burst			
Rank	References	Strength	Begin	End	Duration
1	McDonald LC, 2018, CLIN INFECT DIS, V66, P0 [34]	52.81	2019	2021	
2	Costello SP, 2019, JAMA-J AM MED ASSOC, V321, P156[19]	43.46	2019	2021	
3	Routy B, 2018, SCIENCE, V359, P91[36]	38.83	2019	2021	
4	Quraishi MN, 2017, ALIMENT PHARM THER, V46, P479 [37]	36.19	2019	2021	
5	Kao D, 2017, JAMA-J AM MED ASSOC, V318, P1985 [38]	34.68	2019	2021	
6	Kang DW, 2017, MICROBIOME, V5, P0 [27]	34.54	2019	2021	
7	Gopalakrishnan V, 2018, SCIENCE, V359, P97 [42]	33.22	2019	2021	
8	Kootte RS, 2017, CELL METAB, V26, P611 [43]	31.25	2019	2021	
9	Bajaj JS, 2017, HEPATOLOGY, V66, P1727 [44]	28.29	2019	2021	
10	Shono Y, 2016, SCI TRANSL MED, V8, P0 [45]	24.36	2019	2021	

### Clusters of co-cited references

**Fig. 4:**
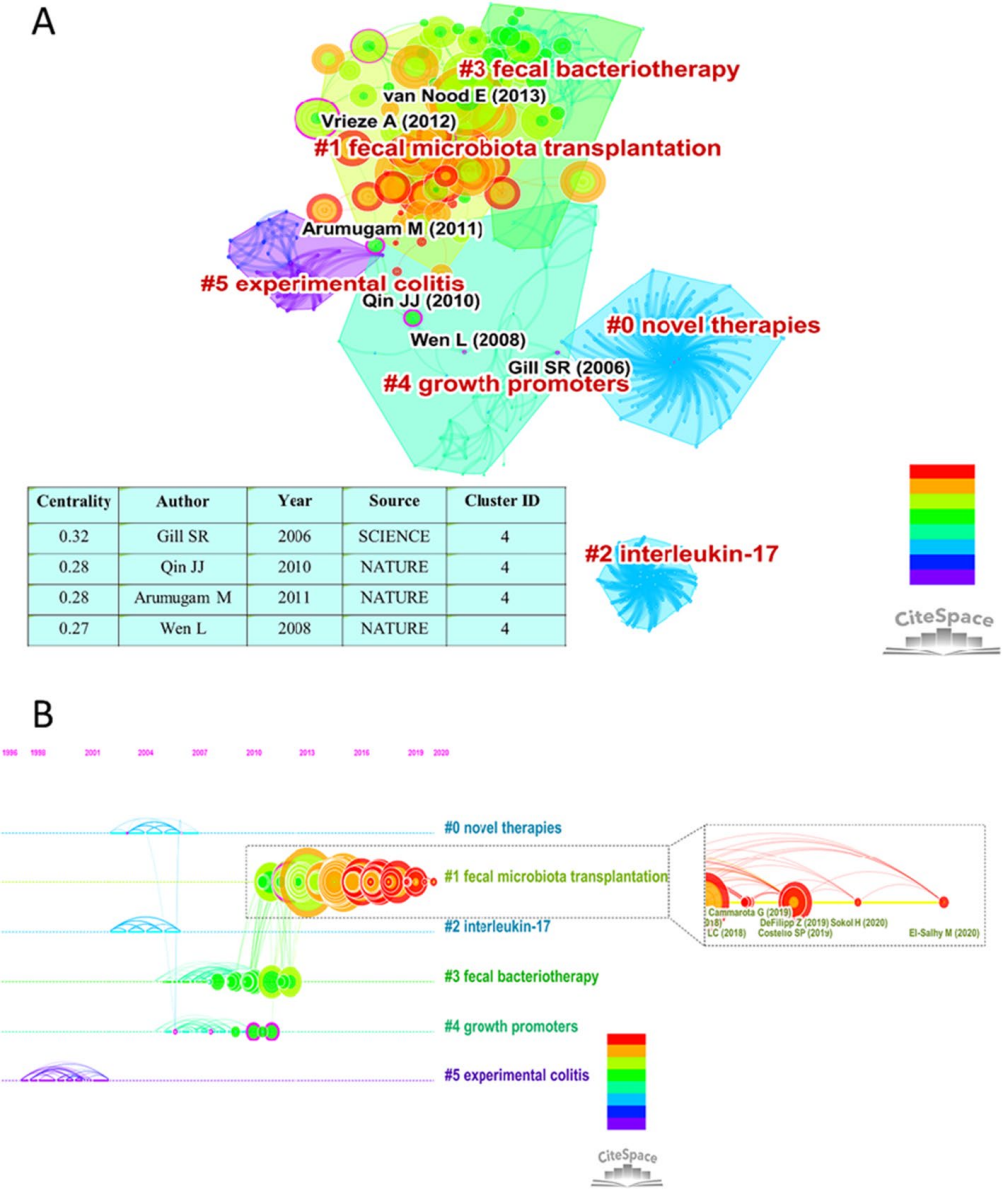
Clusters of co-cited references and visualized timeline for cluster #1. **A** The six largest clusters in the network of co-cited references and top 4 authors with highest centrality in cluster 4. **B** A visualized timeline for cluster #1, and five high-impact contributions with large citation patterns and citation bursts colored in red from 2019 to 2020

Co-citation implies that two related articles (or authors) are cited by the third article (or author) at the same time. A Citespace segments the co-citation network into multiple co-cited reference clusters, keeping references closely connected within the same cluster but loosely connected between distinctive clusters [46]. Figure 4A illustrates the six largest clusters in the network of co-cited references, the details of which are shown in Table 6. The size of each cluster indicates the number of members in it; the larger the cluster, the more representative. The silhouette score, which is an indicator of homogeneity or consistency, can also reflect the quality of a cluster. The silhouette values of homogenous clusters tend to approach 1[46], and most of the clusters in Table 6 are highly homogeneous, which means that the quality of the group is high. LSI, LLR and MI are three different algorithms provided by CiteSpace for extracting clustering labels, in the actual research process, users can use the labels extracted by the LLR algorithm in the visualization network to show the clusters names. The average year of publication of a cluster indicates its recentness. Circles with purple edges show high centrality (which is evaluated by purple edge range of the circle rather than circle size) and act as a bridge between different disciplines [46]. Clearly, cluster #1 and cluster #4 contained the cited references with the highest centrality, and the citation rate of articles in cluster #1 was relatively high, for example, the article with largest circle published by van Nood et al. in cluster #1 and the article with the highest centrality published by Gill et al. [47] in cluster #4 as shown in the table in Fig. 4A. Next, we would focus on these two clusters.

**Table 6:** Top 6 clusters of co-cited references with the highest *K* value

Cluster ID	Size	Silhouette	Label (LSI)	Label (LLR)	Label (MI)	Average year
#0	148	1	Late-breaking news from the “4th international meeting on inflammatory bowel diseases” capri	Capri	Fecal microbiota transplantation	2005
#1	78	0.967	Fecal microbiota transplantation	Versus-host disease	Population health	2014
#2	56	1	role of intestinal subepithelial myofibroblasts in Inflammation and regenerative response in the gut	Regenerative response	Fecal microbiota transplantation	2003
#3	51	0.97	Fecal microbiota transplantation	Fecal bacteriotherapy	Fecal microbiota transplantation	2008
#4	41	0.989	Intestinal microbiota	Vancomycin- resistant enterococcus domination	Fecal microbiota transplantation	2007
#5	29	0.983	Fecal microbiota transplantation	Using fecal Bacteriotherapy	Fecal microbiota transplantation	1999

Clusters are referred in terms of the labels selected by log-likelihood ratio test method (LLR)

*LSI* latent semantic index, *MI* mutual information

Cluster #1 on FMT, which had an average publication year of 2014, was the most recently formed cluster, and it contained numerous nodes with red rings of citation bursts [32]. We selected the 5 most cited references in this cluster and 5 citing articles, as listed in Table 7. The most cited article in this cluster was an initial randomized trial that compared FMT with vancomycin in the treatment of RCDI [14] and demonstrated that for the treatment of RCDI, an infusion of donor feces was more effective than the usage of vancomycin. The second most cited reference came from Moayyedi et al. [17], who found that FMT induced significantly higher remission rates than placebo in active UC patients, with no discrepancy in terms of adverse events. Cluster #1 consisted of 301 co-cited references. The five selected citing articles were all reviews, and two articles were published in 2019. Unlike previous articles, the application of FMT in these two articles was not limited to CDI, it could also be used in HE and cardiometabolic syndrome [15, 50]. Furthermore, we created a visualized timeline for cluster #1, as shown in Fig. 4B. We selected five high-impact contributions with large citation patterns and citation bursts colored in red from 2019–2020; among them, three articles were RCTs [19, 52, 53], and the remaining two were case reports and reviews [54, 55]. DeFilipp et al. described that in two independent clinical trials, extended broad-spectrum β-lactamase (ESBL)-producing Escherichia coli bacteremia occurred in two patients after they received FMT, both from the same fecal donor, and one of the patients died. Therefore, there is a need for enhanced donor screening to limit the spread of microorganisms that may lead to adverse infectious events and continued vigilance to determine the indications for and risks of FMT in different patient groups.

**Table 7:** Top 5 cited references and citing articles of Cluster #1

	Cluster #1 Fecal microbiota transplantation	
Freq	Cited references (author/year/source/vol/page)	Citing articles (author/year/title)
474	van Nood E/2013/NEW ENGL J MED/V368/P407 [14]	Brandt, Lawrence J/2013/An overview of fecal microbiota transplantation: techniques, indications, and outcomes [48]
335	Moayyedi P/2015/GASTROENTEROLOGY/V149/P102 [17]	Kelly, Colleen R/2015/Update on fecal microbiota transplantation 2015: indications, methodologies, mechanisms, and outlook [49]
255	Paramsothy S/2017/LANCET/V389/P1218 [18]	Leshem, Avner/2019/Fecal microbial transplantation and its potential application in cardiometabolic syndrome [50]
241	Rossen NG/2015/GASTROENTEROLOGY/V149/P110 [16]	Allegretti, Jessica R/2019/The evolution of the use of fecal microbiota transplantation and emerging therapeutic indications [15]
214	Cammarota G/2017/GUT/V66/P569 [39]	Brandt, Lawrence J/2017/Fecal microbiota therapy with a focus on clostridium difficile infection [51]

Cluster # 4 on growth promoters had four articles with high centrality, and among them, three articles were published in Nature. Qin et al. [56] depicted Illumina-based macrogenome sequencing, assembly and characterization of 3.3 million irredundant microbial genes from the sequences of 576.7 gigabases derived from fecal samples of 124 European individuals. Arumugam et al. [57] integrated 22 newly sequenced personal fecal macrogenomes from four different countries with previously published datasets and they identified three not nation or continent specific robust clusters. The work of these two articles can be regarded as the basis for FMT. The third article demonstrated that gut microbes interact with the innate immune system as a key epigenetic factor in altering type 1 diabetes (T1D) susceptibility [58].

### An alluvial flow visualization of highly co-cited references

**Fig. 5:**
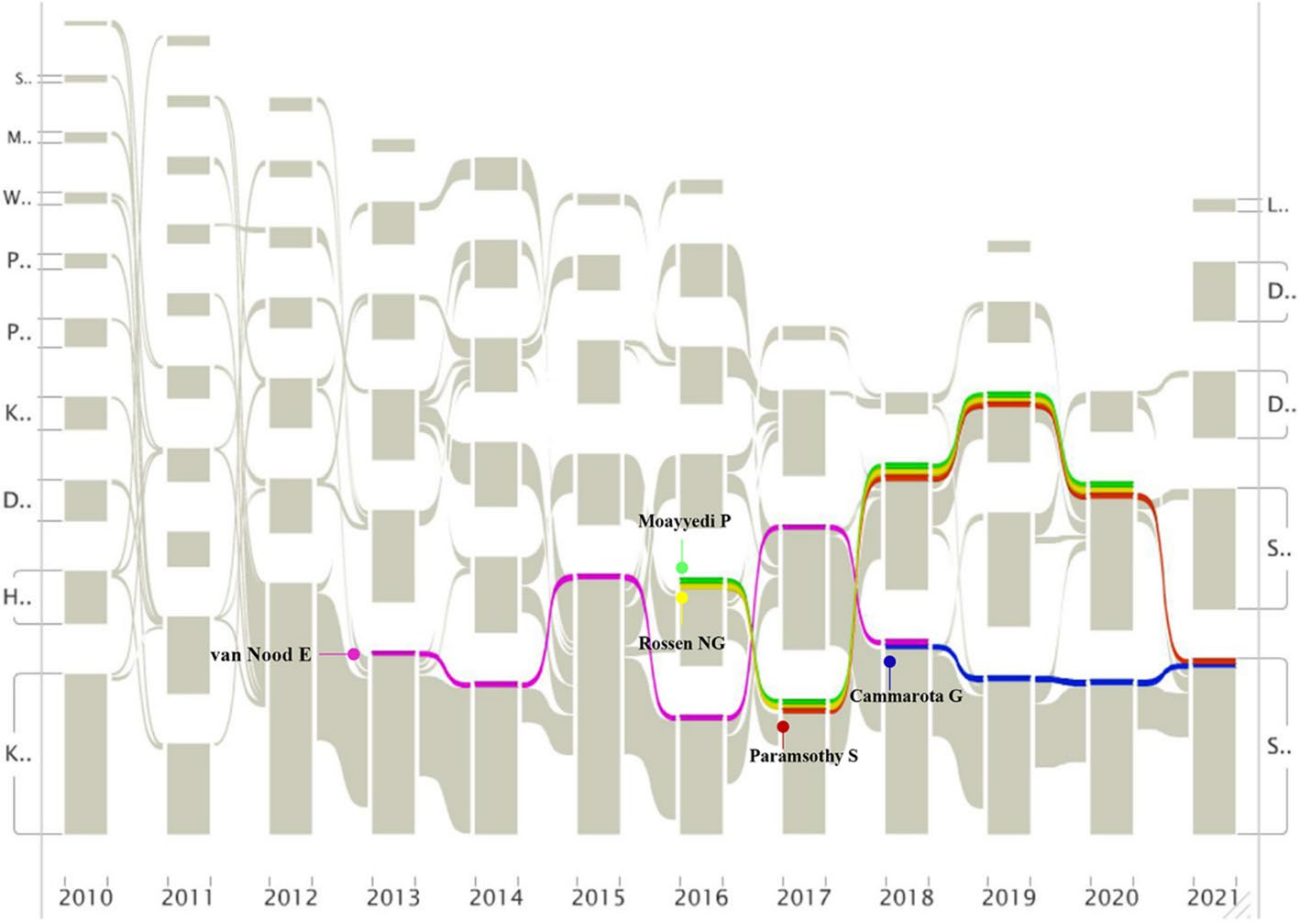
The alluvial flow map of the top 5 co-cited references. Each line represented a study, and colored and continuous lines referred to articles that had been consistently cited

Alluvial flow diagrams aim to reveal time patterns in evolutionary networks [59]. To observe the citation changes of top 5 cited references in cluster 1 intuitively, we generated an alluvial map. First, the data were retrieved from CiteSpace, and networks of co-cited references were generated. Because the top 5 cited references were all published in 2010–2021, the networks exported into the alluvial generator (https://www.mapequation.org/apps/AlluvialGenerator.html) were within this timespan. Nodes in the import network that had the longest presence are highlighted by coloring the flows they form. As shown in Fig. 5, the article with the longest presence was published by van Nood et al. and its presence lasted for six years but did not continue until 2021. Two articles from Paramsothy et al. and Cammarota et al. presented a noninterrupted presence to 2021. The former evaluated the efficiency and safety of FMT in patients with UC in a double-blind randomized trial which demonstrated that in patients with active UC, intensive drug administration, multiple donors, FMT induced clinical remission and endoscopic improvement was associated with different microbial changes. The latter is a European consensus conference on FMT, which provided statements on FMT indications; donor selection; clinical management and basic requirements for implementing an FMT center. These most cited articles mentioned above in cluster 1, especially the presence of which continued until 2021, can represent research hotspots of FMT to some extent.

### Analysis of thematic evolution and keyword citation bursts

**Fig. 6:**
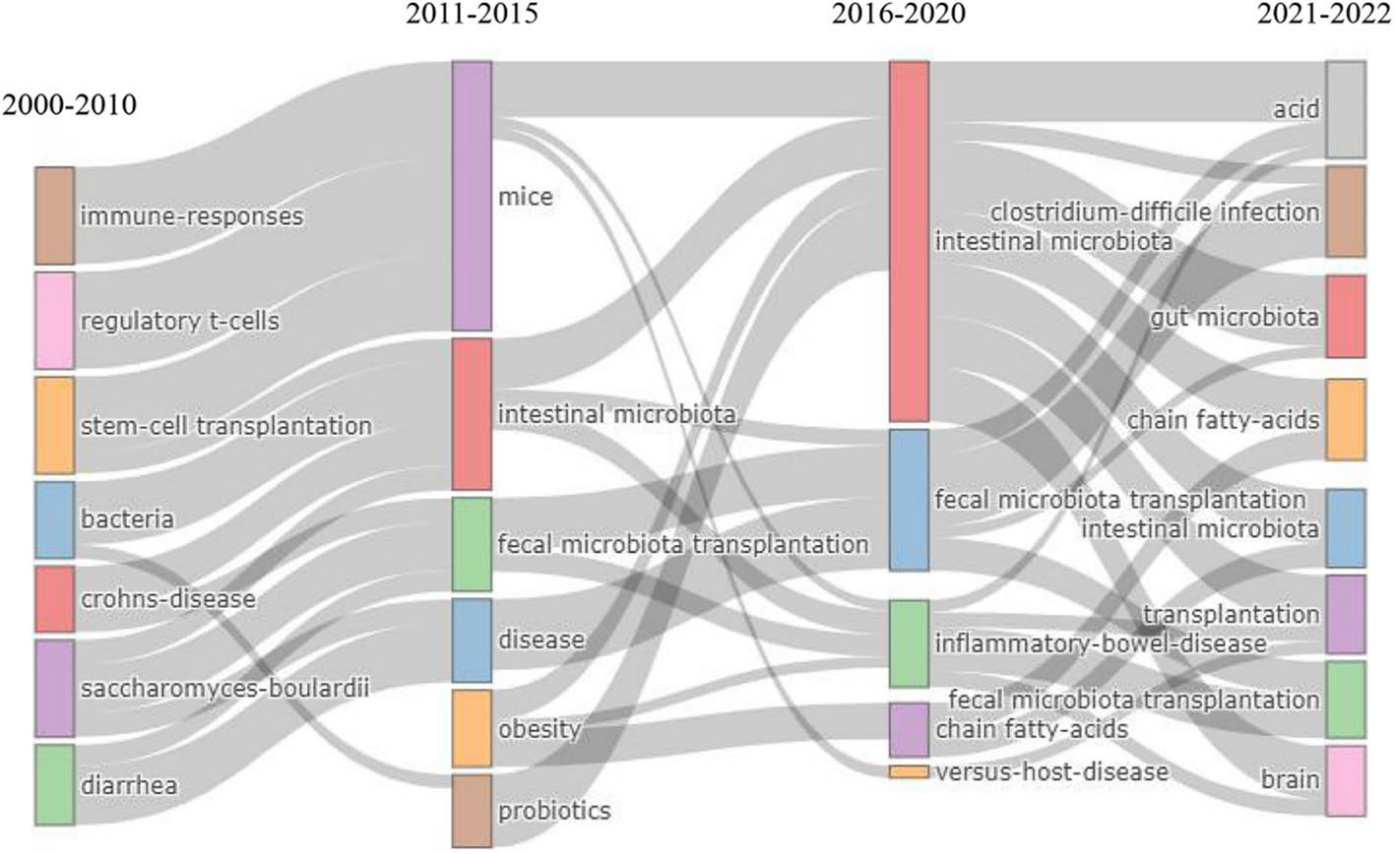
Sankey diagram of keywords evolution of fecal microbial transplantation

Keywords can summarize research topics, through the analysis of keywords, we can understand the research hotspots in specific fields. In the present work, the terms from “keywords Plus” based on the Web of Science Core Collection database and keyword citation bursts can be considered emerging topics within this domain. Therefore, thematic evolution analysis of the keywords was performed. Figure 6 clearly shows the research progress in this field. The initial stages of research on FMT involved “immune responses”, “stem-cell transplantation”, and “bacteria”. However, as this area of research matured, the main research hotspots of FMT gradually changed to “obesity”, “probiotics”, and “inflammatory bowel disease”, among others. Recently, the topic of gut microbial metabolites such as “chain fatty acid” and “brain-gut axis” has gradually attracted the attention of scholars. Furthermore, we conducted a keyword citation burst analysis. The top 10 keywords with the most recent bursts are shown in Table 8, among them, “insulin sensitivity” and “*Akkermansia muciniphila*” both with the strongest citation bursts, indicating that FMT may play an essential role in improving insulin sensitivity and then treating diabetes. Meanwhile, with a spurt of progress in science technology, the role of specific strain in disease such as *Akkermansia muciniphila* have been confirmed by analyses, which can provide new ideas and therapeutic targets for the treatment of clinical diseases.

**Table 8:** Top 10 keywords with the strongest citation bursts during 2018–2021

		Citation burst			
Rank	Keywords	Strength	Begin	End	Duration
1	Quality of life	5	2018	2019	
2	Trial	4.81	2018	2019	
3	Guideline	4.77	2018	2019	
4	Frozen	4.45	2018	2019	
5	Insulin sensitivity	9.23	2019	2021	
6	*Akkermansia muciniphila*	6.68	2019	2021	
7	Gut microbiome	5.36	2019	2021	
8	Induction	5.06	2019	2021	
9	Immunity	4.92	2019	2021	
10	Association	3.81	2019	2021	

## Discussion

### General information

In this study, we analyzed the major knowledge domains and emerging trends in FMT by using bibliometrics. The results showed that the number of annual publications on FMT presented an upward trend and is currently in a stage of rapid expansion. The analysis of active countries and authors showed that the US is not only the most productive country, but also formed close cooperation networks with many other countries, which means that the US is the premier country for FMT research, meanwhile, the University of Minnesota System and University of Michigan which were located in the US also showed higher centrality. China was the second most productive country, Nanjing Medical University was the main representative Chinese institution. Khoruts A, a gastroenterologist at the University of Minnesota, co-author of the first detailed operational guide to FMT, was the author with the most publications and the highest H-index.

The dual-map overlay of journals intuitively reflect the discipline distribution and application areas of FMT. Cited reference burst analysis and alluvial flow visualization can help us find landmark articles for FMT among the thousands of studies. The results of Table 4 and Fig. 5 show that, van Nood et al., who was the first to demonstrate that FMT is generally safe and highly effective in the treatment of RCDI in RCT, making the article became the strongest and most lasting references during 2013–2018.However, strong side effects had been reported after FMT in 2019 [55], perhaps it was the reason for not continuing of the article by van Nood et al. in the last two or three years. Except for the European consensus conference published by Cammarota et al., the remaining articles were RCTs on UC, which indicates that UC may be a new indication for FMT.

### Frontier and hotspot analysis

The analysis of references citation bursts in recent years may reflect developments in specific fields. Table 5 and Fig. 4B show articles with the most recent bursts and high-impact contributions from 2019 onward. Their main content can be summarized as follows.

The treatment efficacy of FMT in CDI has already been proven. However, it is uncertain whether the preparation and mode of administration of FMT affect clinical efficacy, so this has become the focus of discussion recently. A systematic review with a meta-analysis showed that FMT is an effective treatment for RCDI, regardless of the preparation method or delivery approach [37]. A randomized controlled trial showed comparative efficacy of FMT observed in subjects receiving fresh or frozen fecal products from the same donors. The efficacy of the freeze-dried product was slightly reduced compared to that of the fresh product but was similar to other treatments in terms of microbial recovery 1 month after FMT [56]. In addition, Costello et al. [19] proved that in patients with mild to moderate UC, treatment with anaerobically prepared donor FMT for 1 week was preferred to result in remission at 8 weeks compared to treatment with autologous FMT. Kao et al. [39] demonstrated that in adults with RCDI, oral capsule FMT was not inferior to colonoscopy in preventing recurrent infections over 12 weeks. Taken together, the correlation between the preparation and delivery method of FMT and clinical efficacy has become a research hot topic, and more convenient methods may appear in the near future, for example, oral FMT [60].

### Possible future directions and areas for FMT

With the development of FMT, a number of emerging research areas are becoming the subject of interest for researchers. Keywords represent the research theme and core content of the literature. The analysis of thematic evolution and keyword citation bursts showed that some items had higher burst strengths. Figure 6 and Table 8 show that flora metabolites such as “short-chain fatty acids”, specific strains such as “*Akkermansia muciniphila*”, “brain” and metabolism-related terminology such as “inulin sensitivity” are emerging as research hotspots.

### Microbial metabolites—short-chain fatty acids

SCFAs are carboxylic acids with less than 6 carbon atoms and are the most commonly studied metabolites of the intestinal microbiome [61]. Over 95% of the SCFAs produced in the intestine are acetate, propionate, and butyrate, although small amounts of valerate, isovalerate, propionate, hexanoate, isohexanoate, succinate, isobutyrate, and caproate are also found [62–64]. SCFAs are involved in many different host physiological processes, including gastrointestinal function [65], the regulation of blood pressure [66], circadian rhythms [67], and innate and adaptive immune regulation [68]. Therefore, the occurrence of various diseases is accompanied by altered fecal short-chain fatty acid content. The results of our bibliometric analysis also showed that targeted dietary SCFAs may be a mechanism to alter immune profiles, promote immune tolerability, and enhance glucose control for the therapy of T1D [69]. In addition, patients with IBD and PD all exhibited abnormal of SCFAs level in fecal [70–72]. All the above indicated that, SCFAs play a major role in metabolic and neurological disorders.

### Specific strain of bacteria—Akkermansia muciniphila

*Akkermansia muciniphila* is a Gram-negative and strictly anaerobic bacterium that was first isolated from the human face in 2004 [73]. *Akkermansia muciniphila* belongs to the Verrucomicrobia phylum and was found to be abundant in the human intestine [74, 75]. Growing evidence demonstrates that *A. muciniphila* can effectively improve metabolic disorders and is therefore regarded as a promising “next generation of beneficial microorganisms” [76]. Our findings also indicated that the abundance of *A. muciniphila* is indeed associated with many diseases, metabolic diseases such as type 2 diabetes (T2D) [77], obesity [78–80], Binge eating disorder (BED) [81] and psoriasis [82], which indicates that microecological agents may become an important therapeutic approach in the future.

### Microbiota–gut–brain axis

The existence of the “brain–gut axis” was first identified in the mid-nineteenth century by Doctor William Beaumont [83], who found that when his patient was upset or annoyed, it greatly affected the digestive rate, suggesting that the emotional state of an individual can affect their digestion, i.e., the presence of the brain–gut axis. With the emergence of brain imaging in the 1980s, a comprehensive interpretation of the bidirectionality of this axis appeared. Even though the microbiota–gut–brain axis is an emerging concept, it is gradually being accepted that microbial residents can exert considerable influence on host performance [84–87], and this axis has received increasing attention in the field of investigating the biological and physiological basis of psychiatric, neurodevelopmental, age-related and neurodegenerative diseases [88]. Therefore, some probiotics are widely used in the clinic. Several studies have shown that probiotics are effective in improving mood and cognition and relieving stress and anxiety [89–92]. In addition, researchers have shown that the microbiota–gut–brain axis is associated with neurological diseases and disease processes, including autism spectrum disorder [27, 93], major depressive disorder [94], anxiety [95, 96] and schizophrenia [97]. The above results from preclinical and clinical studies have opened up the potential for targeting the gut microbiota in the treatment of psychiatric and neurological disorders.

## Conclusions

FMT has significant research value and promising applications. The number of studies on FMT generally shows an increasing trend. The US and China are the leaders in this research. Among the research institutions, Harvard Medical School is the institution with the most articles, while its centrality is less than 0.1, which indicated that its collaboration with other institutions should be strengthened. Khoruts A is an excellent pioneer in the field of FMT. Most publications on FMT have been cited in influential international journals, indicating that FMT has received widespread attention. Current research on FMT is focused on microbiota metabolites, specific strains of bacteria, the microbiota–brain–gut axis and the role of FMT in other diseases, which will be the focus of future research.

Although following the principles of certain bibliometrics and comprehensive analysis strategies, there are some inevitable limitations to our current study. First, only articles and reviews from the Web of Science Core Collection databases in a particular time period were used, so this may lead to literature and publishing bias. Second, our search strategy included only some specific terms relating to FMT, therefore, the retrieved publications may contain false positives and false negatives. Finally, to date, there is a lack of adherence to ethically accepted international standards in bibliometric analysis, and the constraints of analytical tools may provide subjective views of an individual's work and contributions. Despite these limitations, due to the sufficient number of documents collected in this analysis, our bibliometric showed the advanced countries, institutions and pioneers in studying FMT and summarized research frontiers, hotspot and landmark articles in the development of FMT adequately. We convinced that what we found about FMT studies can provide a complete picture and contributes to current research and future directions in the field.

## Methods

### Data source and search strategy

Data were derived from the Web of Science Core Collection which covers the largest and most comprehensive literature and were downloaded on January 3, 2022. The retrieval formula was defined as TS = (fecal microbial transplantation) OR TS = (intestinal microbiota transplantation) OR TS = (fecal bacteriotherapy). According to our search strategy, the literatures before 2000 has little relevance to FMT, so we determined the search time range from January 2000 to December 2021. The search resulted in 4042 records being identified, and 898 irrelevant articles such as “meeting abstracts”, “book chapter”, “editorial materials”, “early access”, “proceeding paper”, “correction” and “letters” were excluded. Finally, 3144 documents include “article” and “review” were exported into a form of all records and references, saved as plain text files and then saved in download_txt format.

## Data Availability

The initial contributions discussed in the study are included in the article material, and further queries can be referred directly to the corresponding author.

## Abbreviations

SCFAs: Short-chain fatty acids
LPS: Lipopolysaccharide
FMT: Fecal microbial transplantation
RCTs: Randomized controlled clinical trials
RCDI: Recurrent *Clostridioides difficile* infection
IBD: Inflammatory bowel disease
UC: Ulcerative colitis
HE: Hepatic encephalopathy
PSC: Primary sclerosing cholangitis
GIT: Gastrointestinal tract
US: United States
IBS: Irritable bowel syndrome
CNS: Central nervous system
ENS: Enteric nervous system
CD: Crohn’s disease
COVID-19: Coronavirus disease 2019
SARS-CoV-2: Severe acute respiratory syndrome coronavirus 2
T2D: Type 2 diabetes
BED: Binge eating disorder
